# Rectus femoris hyperreflexia contributes to Stiff-Knee gait after stroke

**DOI:** 10.1186/s12984-020-00724-z

**Published:** 2020-08-26

**Authors:** Tunc Akbas, Kyoungsoon Kim, Kathleen Doyle, Kathleen Manella, Robert Lee, Patrick Spicer, Maria Knikou, James Sulzer

**Affiliations:** 1 School of Engineering and Applied Sciences, Harvard University, Cambridge, MA USA; 2 Walker Department of Mechanical Engineering, The University of Texas at Austin, Austin, TX USA; 3 University of St. Augustine for Health Sciences, Austin, TX USA; 4 St. David’s Medical Center, Austin, TX USA; 5 Seton Brain and Spine Institute, Ascension Texas, University of Texas at Austin, Austin, TX USA; 6 Graduate Center of the City University of New York and Physical Therapy Department, College of Staten Island, New York, NY USA

**Keywords:** Post-stroke gait, Stiff-knee gait, Spasticity, Hyperreflexia, H-reflex

## Abstract

**Background:**

Stiff-Knee gait (SKG) after stroke is often accompanied by decreased knee flexion angle during the swing phase. The decreased knee flexion has been hypothesized to originate from excessive quadriceps activation. However, it is unclear whether hyperreflexia plays a role in this activation. The goal of this study was to establish the relationship between quadriceps hyperreflexia and knee flexion angle during walking in post-stroke SKG.

**Methods:**

The rectus femoris (RF) H-reflex was recorded in 10 participants with post-stroke SKG and 10 healthy controls during standing and walking at the pre-swing phase. In order to attribute the pathological neuromodulation to quadriceps muscle hyperreflexia and activation, healthy individuals voluntarily increased quadriceps activity using electromyographic (EMG) feedback during standing and pre-swing upon RF H-reflex elicitation.

**Results:**

We observed a negative correlation (*R* = − 0.92, *p* = 0.001) between knee flexion angle and RF H-reflex amplitude in post-stroke SKG. In contrast, H-reflex amplitude in healthy individuals in presence (*R* = 0.47, *p* = 0.23) or absence (*R* = − 0.17, *p* = 0.46) of increased RF muscle activity was not correlated with knee flexion angle. We observed a body position-dependent RF H-reflex modulation between standing and walking in healthy individuals with voluntarily increased RF activity (*d* = 2.86, *p* = 0.007), but such modulation was absent post-stroke (*d* = 0.73, *p* = 0.296).

**Conclusions:**

RF reflex modulation is impaired in post-stroke SKG. The strong correlation between RF hyperreflexia and knee flexion angle indicates a possible regulatory role of spinal reflex excitability in post-stroke SKG. Interventions targeting quadriceps hyperreflexia could help elucidate the causal role of hyperreflexia on knee joint function in post-stroke SKG.

## Introduction

Stiff-Knee gait (SKG) is one of the most common gait disabilities following stroke. SKG is defined as reduced knee flexion [30] during the swing phase. Those with SKG have joint pain [16], energy inefficiency due to compensatory motions [9, 36, 38] and increased risk of falls [3]. Post-stroke SKG has been attributed to overactivity of rectus femoris (RF) muscle [2, 13, 14] and decreased activity of ankle plantar flexors and iliopsoas that generate knee flexion moment [23, 29]. Quadriceps muscle overactivity is the most widely accepted cause of SKG [18, 33, 42]. To this end, Botulinum toxin (Botox) injections that block acetylcholine release in the femoral nerve show modest improvements in knee flexion [34, 35, 39], suggesting that rectus femoris (RF) reflex excitability contributes to SKG. However, the cause of excessive RF muscle activity is unclear. One hypothesis is that increased quadriceps activation could be achieved voluntarily to improve stability during the stance phase, but then fails to relax during the pre-swing phase [18, 33]. RF overactivity could additionally be explained as spasticity in the form of reflex hyperexcitability and lack of reciprocal inhibition.

Accumulating evidence suggests that reduced knee flexion in post-stroke SKG depends on pathological modulation of spinal reflex loops. For example, Lewek et al. [27] found increased quadriceps short-latency reflex excitability following hip extension perturbations in post-stroke individuals. The degree of hyperexcitability was correlated to knee flexion angle, suggesting that altered involuntary responses could play a role in diminished knee flexion during the swing phase of post-stroke SKG. Others found that hip abduction perturbations elicited abnormally coordinated RF activation, suggesting a role of abnormal reflex-mediated coordination in post-stroke gait [12]. Using a custom robotic actuator [41] to perturb knee flexion during pre-swing in individuals with post-stroke SKG [40], we observed a sharp knee extension velocity following initial increased knee flexion angle during swing. However, no such reaction was found during steps where the assistance was temporarily removed, or in a baseline period before the assistance was applied, indicating that the knee extension was induced by the robotic assistance. The knee extension was preceded by increased RF electromyographic (EMG) activity within a short latency following the perturbation, and further musculoskeletal modeling analysis showed a correlation between RF fiber stretch velocity and RF activity [1]. Taken together, this evidence points to RF reflex hyperexcitability influencing knee flexion in post-stroke SKG. Representative altered reflex pathways in post-stroke include reduced gait phase-dependent modulation [8, 21] and changes in presynaptic inhibition [11]. However, their relation to SKG has yet to be determined.

The objective of this study was to characterize the relation of hyperreflexia to knee flexion in post-stroke SKG. We investigated reflex excitability via the monosynaptic H-reflex with well-established neuronal pathways [20]. H-reflexes are a reliable and consistent probe in identifying the altered neuronal pathways [28]. We examined the modulation of monosynaptic RF H-reflexes during standing and walking in people post-stroke compared to healthy control subjects in order to establish the extent to which reflex modulation is related to the knee flexion angle after stroke. In order to determine the possibility that hyperreflexia is a byproduct of improper timing of RF activity, we compared our results to healthy controls with volitionally up-regulated RF activity during pre-swing. We hypothesized that RF H-reflex hyperexcitability during standing and walking in pre-swing phase in people with post-stroke is associated with decreased knee flexion during swing phase in SKG compared to healthy controls. Characterization of the role of hyperreflexia in SKG and other neuromuscular disorders could help to design targeted treatments for hyperreflexia including but not limited to operant H-reflex conditioning training [43].

## Methods

### Subjects

Ten hemiparetic individuals at least 6 months post-stroke (8 M / 2 F, 54 ± 10 years mean ± SD, Table 1) able to walk for 20 min or more at a treadmill speed of 0.2 m/s participated in the study. All participants provided their written informed consent according to the Declaration of Helsinki while all experimental procedures were approved by the Institutional Review Board of the University of Texas at Austin. Exclusion criteria included: multiple strokes, cerebellar damage, or lower limb musculoskeletal injury. We required at least 2 months since the last spasticity medication. Post-stroke participants whose knee range-of-motion (ROM) was at least 20° less on the affected side compared to the unaffected side were qualified as SKG [40]. Ten healthy subjects (6 M/4 F, 35 ± 13 years mean ± SD, Table S1) with no musculoskeletal injuries also participated in the study.

**Table 1:** Participant characteristics with post-stroke Stiff-Knee gait

Subject no	Age (yrs)	G	Side	W (kg)	Post (yrs)	Med.	Botox	AFO/ Cane^a^	Walking Speed (m/s)	Peak Knee flexion (degrees)
1	41	M	L	100	3	–	No	–	0.5	26.3
2	70	M	R	86	4	Baclofen	No	AFO	0.35	6.9
3	63	M	R	81	1.5	–	Yes	Cane	0.5	19.5
4	55	M	L	110	8	Baclofen	Yes	Cane	–	–
5	45	M	L	76	2.5	–	No	AFO	0.35	11.8
6	59	M	R	83	1	–	Yes	–	0.3	9.9
7	53	M	R	65	3	–	Yes	–	0.3	12.6
8	53	M	R	77	1	–	Yes	–	0.4	15.6
9	41	F	R	64	7	–	No	–	0.5	27.7
10	61	F	L	63	1	–	No	Cane	0.22	9.5
Mean	54.1			80.5	3.2				0.38	15.5
SD	9.62			15.4	2.5				0.10	7.5

*G* gender, *W* weight, *Post* years post-stroke, *Meds* taking Baclofen, *AFO/Cane* use of an ankle–foot orthosis or cane, *M* male, *F* female

^a^All ankle foot orthoses (AFOs) worn by the participants were hinged versions. “Walking Speed” refers to each participant’s maximum comfortable walking speed on treadmill. Subject 4 was only able to walk overground and thus walking speed and peak knee flexion angle measures were not collected

### Data collection

All subjects walked on an instrumented split-belt force treadmill (Bertec, Columbus, OH), during which ground reaction forces were recorded. Lower limb kinematic data were acquired via an inertial motion capture (IMU, Xsens, Enschede, Netherlands). Surface electromyographic (EMG) activity (Bortec, Calgary, AL) was recorded from adductor longus (AL), gluteus medius, *(GMed), tensor fasciae latae (TFL)*, gluteus maximus (GMax), medial hamstrings (MH), RF and vastus medialis (VM) muscles. Lower limb kinematics were collected at 60 Hz, whereas the other data were collected at 1 kHz.

**Fig. 1:**
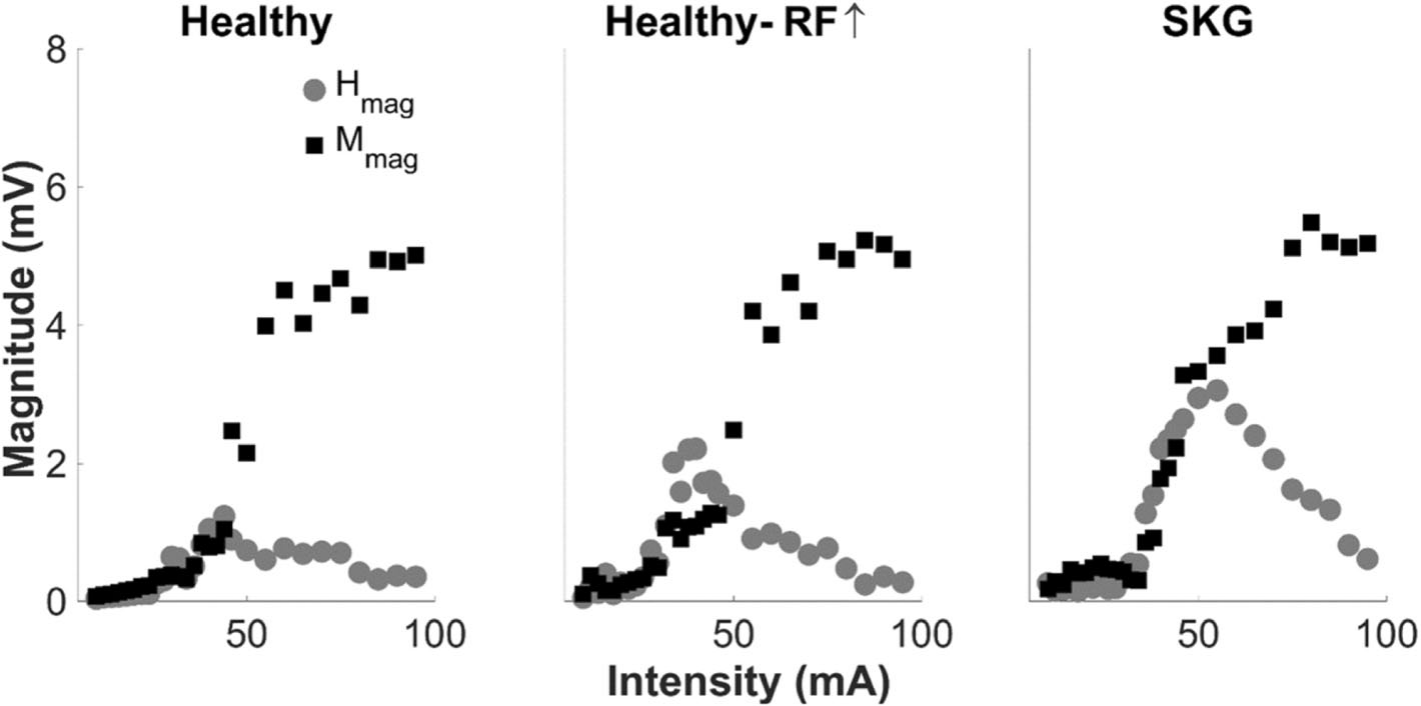
Representative H-reflex recruitment curves for each group. Compared to the H-reflex recruitment curve for a healthy baseline representative (left), there exist increased RF H-waves in both the healthy representative with increased voluntary RF contraction (RF↑, middle) and in the participant with post-stroke SKG (right). The RF↑ during recruitment was achieved by providing RF EMG feedback. No substantial change was observed between maximum M-waves (M_max_) between conditions in healthy individuals

Optimal stimulation of the femoral nerve with a constant current stimulator (Digitimer DS7A, Hertfordshire, UK) was established via a monopolar probe electrode to a grid of points covering the femoral triangle [19, 44]. After the optimal stimulation site was established, the monopolar probe electrode was replaced by a permanent electrode (circular 1 cm diameter) under pressure, and the anode electrode (square 5 × 5 cm) was placed over the gluteus maximus skin of the ipsilateral side. The RF H-reflex recruitment curve was assembled during standing by delivering gradually increasing electrical stimulation to the femoral nerve in 2 mA increments between 10-and 46 mA and 5 mA increments between 50 and 90 mA. Two stimulations were recorded at each stimulus intensity. All H-reflexes were elicited every 7 s to counteract homosynaptic depression [32]. Representative RF H-reflex recruitment curves for a healthy control and a participant with post-stroke SKG are illustrated in Fig. 1.

**Fig. 2:**
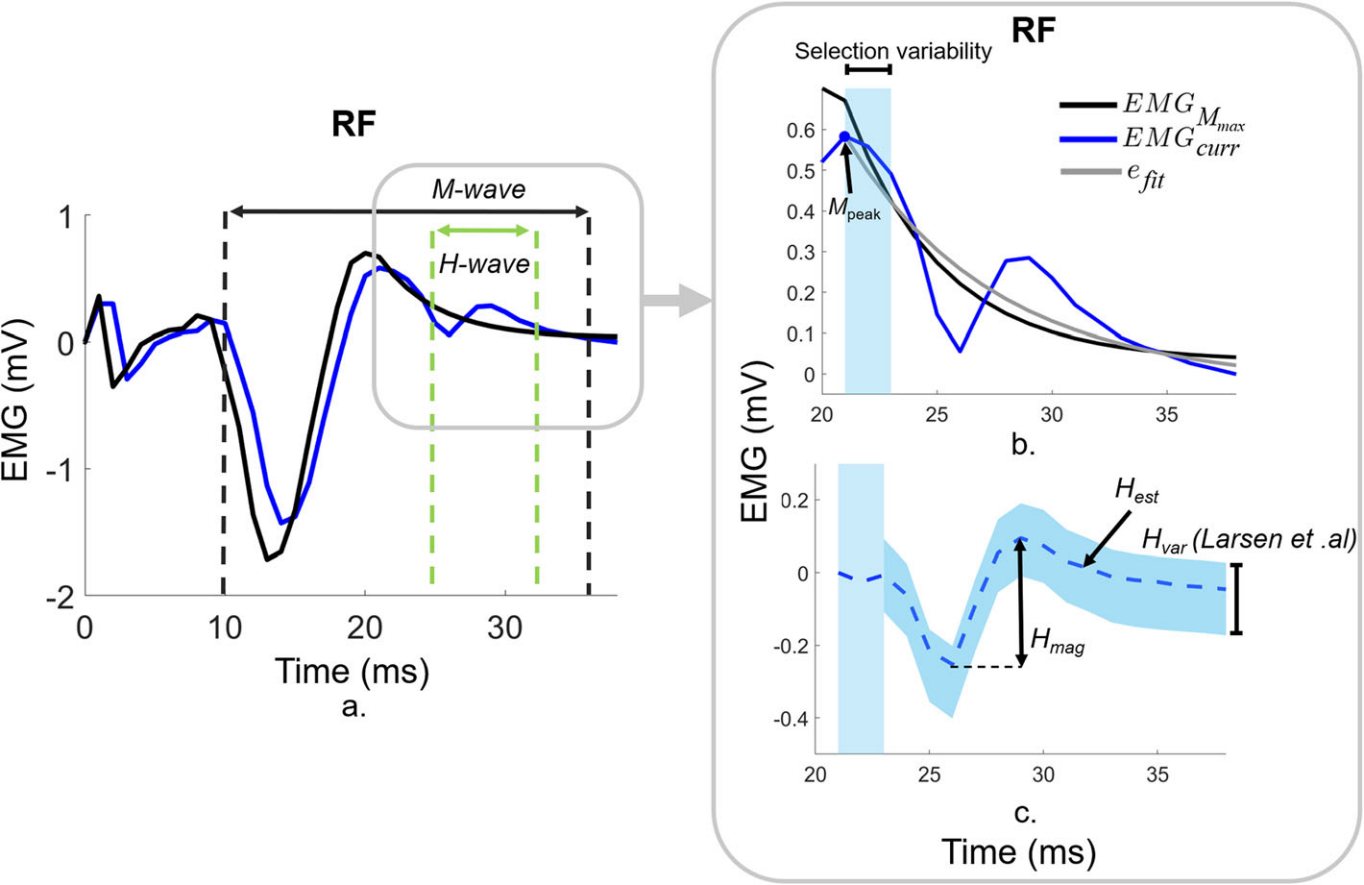
Post-hoc analysis for RF H-reflex magnitude extraction. We modified a previously used method to estimate the H-reflex [24, 25]. EMG response with maximum M-wave (EMG_Mmax_, black) and the current EMG response (EMG_curr_, dark blue) with M-wave and H-wave regions (**a**). An exponential fit (e_fit_, gray) beginning at the peak (M_peak_) of the EMG_curr_ were subtracted from EMG_curr_ instead of the previously introduced procedure [24] using hindmost flank of the EMG_Mmax_ to reduce selection variability indicated by the light blue region (**b**). The estimated H-wave (H_est_) and the variability of the estimated H-waves resulted from hindmost flank selection (H_var_, light blue area, **c**)

The RF H-reflex recruitment curves were problematic when an overlap between the M-wave and H-reflex was present. This issue was counteracted by subtraction of the scaled EMG response of the maximal M-wave (EMG_Mmax_) from the current EMG response (EMG_curr_) [24, 25]. However, this method utilizes a vaguely defined “hindmost flank” of a static EMG_Mmax_, a variable signal compared to a selected EMG_curr_ between participants, to estimate the H-reflex response (Fig. 2), resulting in a variable H-reflex estimate (H_var_) without considering the concurrent M-wave. We modified this approach by selecting the best decaying exponential fit beginning at the peak of the concurrent M-wave, and ending at the zero crossing (Fig. 2b). We then subtracted this best-fit exponential from the measured signal to obtain the H-reflex estimate (H_est_) (Fig. 2c). In addition to avoiding reliance on EMG_Mmax_, this updated method introduces automaticity and robustness to the H-reflex estimation process The corresponding intensities for maximal H-reflexes (H_max_) responses were stored and used in trials.

**Fig. 3:**
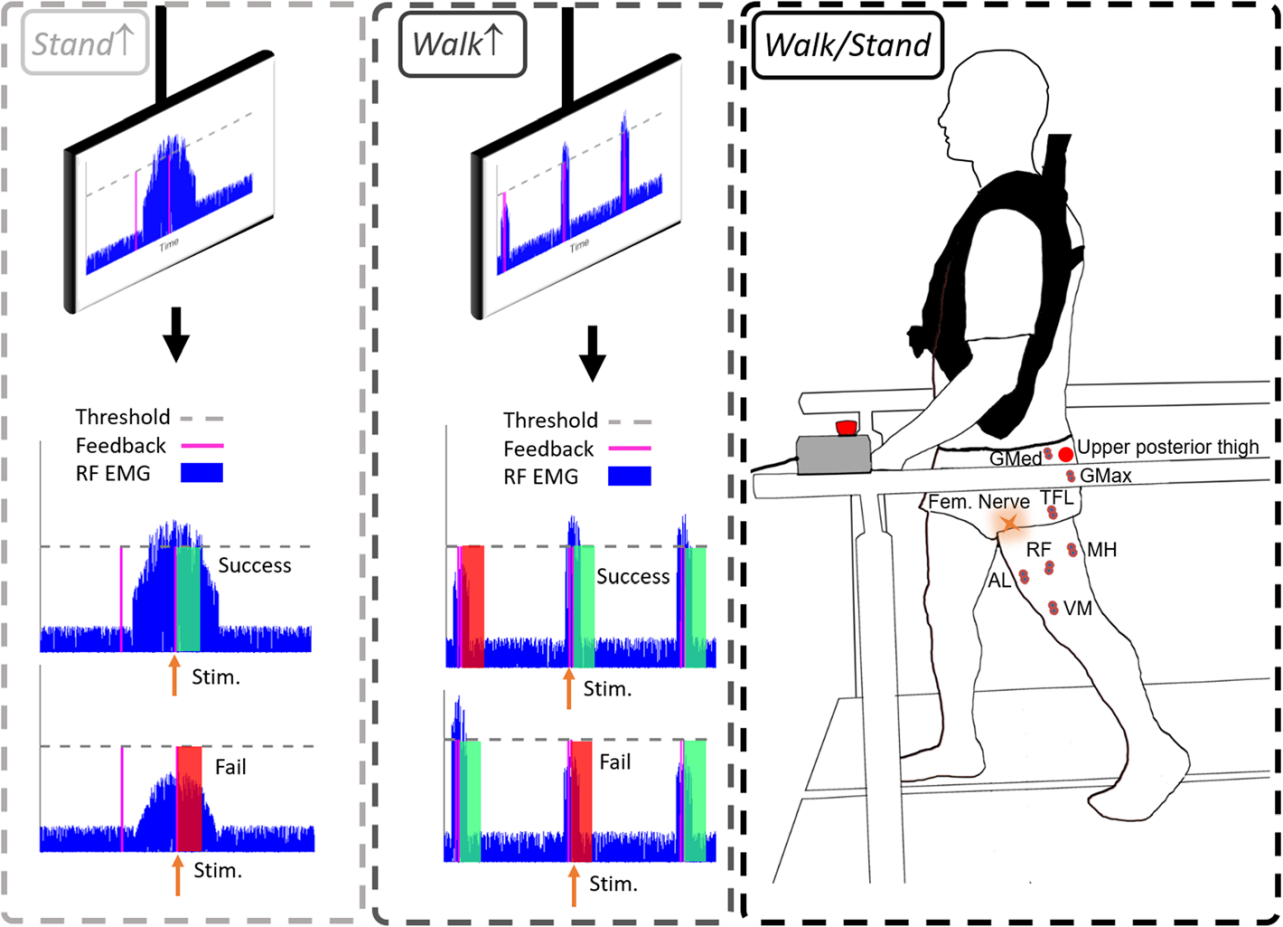
Experimental protocol and RF EMG feedback paradigm. The dashed square boxes indicate the four conditions in experimental protocol, *Stand* (right), during walking at toe-off *Walk* (right), where no visual feedback is provided. In both the *Stand*↑ (left) and *Walk*↑ (middle) conditions, visual feedback of RF EMG is provided for purposes of up-regulation during pre-swing. Participants were instructed to increase RF EMG signal above the threshold value (dashed gray line), 20% of RF maximum voluntary contraction (MVC) following a cue signal in real-time (purple vertical line) with 300 ms and 20 ms offset time prior to stimulation, respectively. For *Walk*↑, the cue was displayed every gait cycle to account for dynamic adaptation during walking. The trials were considered “successful” if the increased rectified RF EMG exceeded the threshold during stimulation (dashed horizontal line) and considered “fail” otherwise

Femoral nerve stimulation was delivered at an intensity that evoked a maximal RF H-reflex (H_max_) during standing (*Stand*), walking at pre-swing (*Walk*), standing with increased RF EMG activity (*Stand*↑), and walking during pre-swing with increased EMG activity (*Walk*↑). These conditions are graphically shown in Fig. 3. For each participant and condition, 25–30 RF H-reflexes were recorded 7 to 9 s apart in pseudo-random order during standing and pre-swing, matching the timing of the knee flexion perturbation in our previous study [40]. The step cycle phases were detected automatically in real-time using the vertical ground reaction force of the affected side from the instrumented treadmill. People maintained balance via supporting handrails, while an upper body harness was worn to prevent falls. Subjects walked at 0.5 m/s, or if not possible, their maximum comfortable walking speed, for 15 min in the walking conditions. The *Stand* and *Walk* H-reflexes were recorded randomly in post-stroke SKG subjects. We did not conduct *Stand*↑ or *Walk*↑ conditions in SKG. For healthy controls, the walking speed was fixed at 0.5 m/s for the walking conditions and all the conditions were randomized. The corresponding H_max_ was normalized to the associated maximal M-wave (M_max_) for comparisons among conditions and groups.

### RF EMG feedback

We recorded RF maximum voluntary contraction (MVC) during isometric contractions in standing. We used this value to compute a 20% RF MVC feedback target for *Stand*↑ and *Walk*↑ conditions in healthy participants (Fig. 3). In *Stand*↑, participants were cued 300 ms prior to stimulation, which helped with improving trial success rate. However, in *Walk*↑, the cue was 20 ms prior, which was determined heuristically in order to both prevent effects on earlier portions of the gait cycle but also to maximize success rate. The participants were instructed to increase their RF EMG signal above the displayed continuous threshold value scrolling horizontally with trial onsets denoted by vertical lines at least 7 s apart (Fig. 3). The trials were considered “successful” when the increased rectified RF EMG exceeded the threshold. The condition continued until 20 successful trials were obtained (Fig. 3 - *Stand*↑). For the walking feedback conditions of the controls, the onset cue occurred when the ground reaction force of the stimulated limb fell below 30% of body weight, representing late pre-swing, displayed for every gait cycle with the same threshold value in standing. We provided stimulation 20 ms after the onset cue to provide consistent visual feedback through the real-time interface for the corresponding subject. The repetitive stimulations were applied during pseudorandom steps, at least 5 s apart from each other, and continued until 20 successful trials were obtained (Fig. 3 - *Walk*↑), the average success rate was 87.8% ± 20.5. Since the subjects were required to adjust their walking in order to achieve increased voluntary RF contraction, an adaptation period without nerve stimulations was implemented until the participants were able to successfully complete ten consecutive steps with voluntary RF contraction.

### Data exclusion

In one post-stroke individual we were not able to successfully elicit a RF H-reflex, while motion data were not recorded from another post-stroke individual due to equipment failure. Therefore, we report H-reflex data for *N* = 10 participants and kinematic data for *N* = 9 of those participants. RF EMG biofeedback data from one healthy participant were rejected due to inability to achieve the task of voluntarily increasing RF EMG response. H-reflex data of one healthy individual was over 2.5 standard deviations above the mean of the remaining healthy subjects and was declared an outlier and removed from further data analysis. In summary, of the 11 post-stroke and 10 healthy individuals recruited and enrolled, one post-stroke individual could not complete the study, kinematic data was unavailable for another post-stroke subject (S4), RF EMG biofeedback data was not included in one healthy subject (H10), and H-reflex data of another healthy subject was rejected as an outlier (H5).

### Statistical analysis

We investigated the hypothesis that post-stroke reflex excitability during pre-swing was related to swing phase knee flexion angle. Further, we hypothesized that increased RF activity does not explain the coupling between RF reflex excitability and knee flexion. Lastly, we hypothesized that context-dependent (i.e. walking and standing) reflex modulation is impaired in stroke compared to healthy individuals. Main outcome measures were the peak-to-peak H-reflex amplitude normalized to the M_max_ and the peak knee flexion angle during swing phase. We used linear-mixed effect models with Tukey post-hoc *t*-tests (α < 0.05). Interactive effects were group (SKG and healthy) and task (i.e. standing or walking), with a random effect of subject. We used peak knee flexion angle as a covariate. The dependent variable was the RF H-reflex normalized amplitude. The correlation between reflex response and peak knee flexion angle was evaluated using the interaction between walking conditions. Pearson correlation coefficients were established between peak knee flexion angle and H-reflex amplitude.

### Post-hoc EMG analysis

We assessed the sensitivity of the H-reflex responses for the given muscle state during standing trials using the correlation between the RF EMG prior to the stimulation and corresponding H-reflex response both for healthy individuals and participants with post-stroke SKG. The raw RF EMG signals were filtered with a fourth-order band-pass Butterworth filter with cutoff frequencies of 20–400 Hz to remove artifacts, rectified, low-pass filtered (fourth-order low-pass Butterworth, 12 Hz) and normalized with respect to the maximum measures collected throughout the walking session excluding the regions of stimulation (throughout the 60 ms starting from stimulation onset). The muscle activity was quantified by the average normalized RF EMG measure within the 120 ms time interval prior to the stimulation.

## Results

**Fig. 4:**
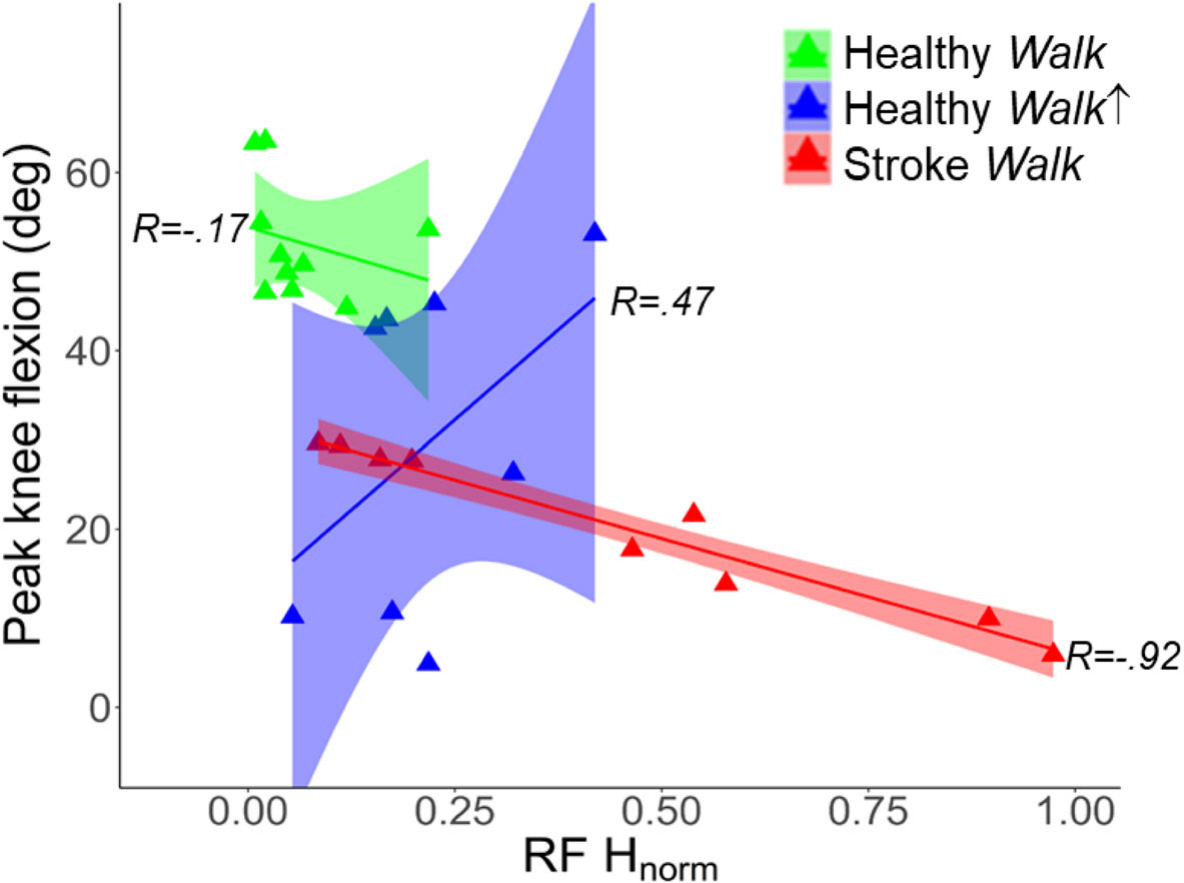
Diminished knee flexion is correlated with increased RF H-reflex in post-stroke SKG. Relations between swing-phase knee flexion angle and RF reflex excitability in post-stroke SKG shows a strong negative effect (red triangles) whereas no significant correlation was observed in healthy *Walk* (green triangles) and healthy *Walk*↑ (blue triangles). The lines and shaded areas indicate the linear regression fits and 95% confidence interval respectively for the corresponding groups

We observed a strong relationship between peak knee flexion angle during swing phase and RF H-reflex in post-stroke SKG (*R* = − 0.92, *p* < 0.001). No correlation was observed for healthy *Walk* (*R* = - 0.17, *p* = 0.46) or healthy *Walk*↑ (*R* = 0.47, *p* = 0.23) conditions, as illustrated in Fig. 4. The correlation between H-reflex amplitude and knee flexion angle SKG was different compared to healthy *Walk* (t = 5.81, *p* = 0.028) and healthy *Walk*↑ (t = 9.33, *p* = 0.011). There was no significant difference observed between healthy walking conditions (t = 0.75, *p* = 0.491).

**Fig. 5:**
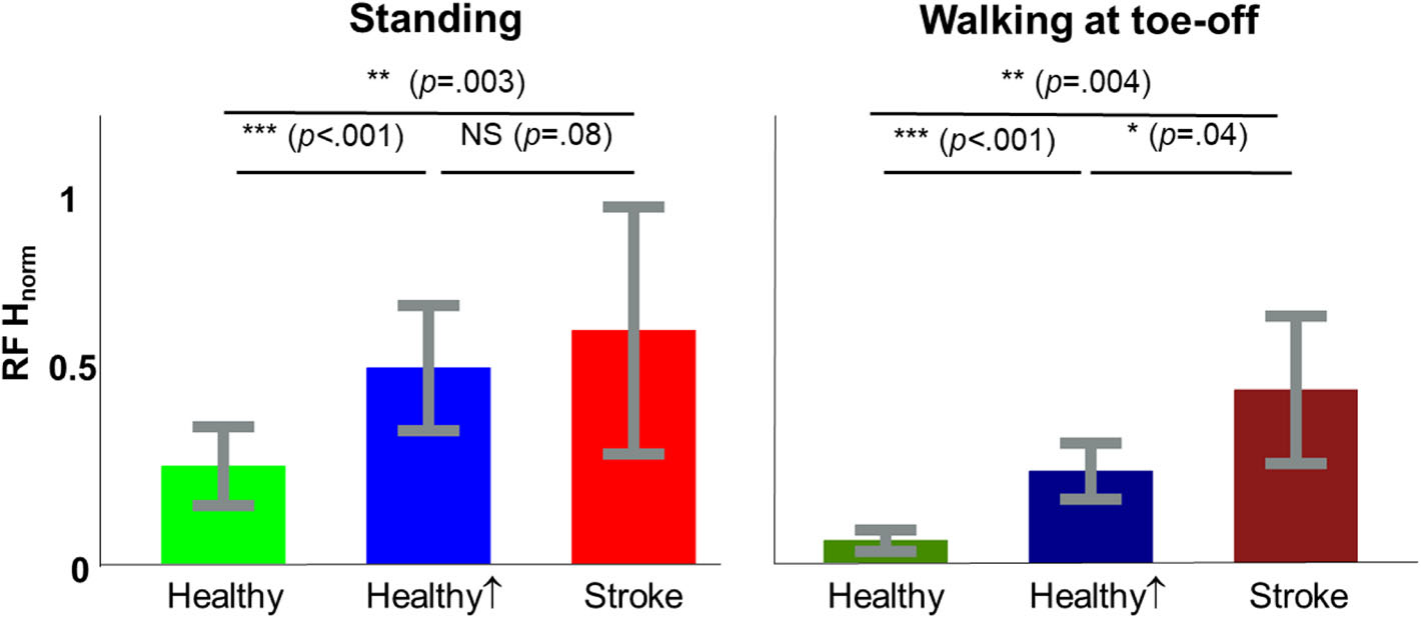
Elevated RF H-reflex in post-stroke SKG is higher than increased voluntary RF contraction. During standing, RF H-reflex response was significantly increased in post-stroke SKG and in healthy individuals with increased voluntary RF contraction compared to healthy baseline. During walking however, RF H-reflex response was increased in post-stroke SKG compared to both healthy baseline and with increased voluntary contraction

During standing, the RF H-reflexes in post-stroke SKG (*N* = 10) trended similarly from those observed in healthy controls in *Stand*↑ (F_(1,326)_ = 3.73, *p* = 0.08, d = .97). Figure 5 shows both the SKG group and healthy *Stand*↑ RF H-reflexes were greater than healthy *Stand* (F_(1,363)_ = 12.3, *p* = 0.003, d = 1.75; F_(1,381)_ = 191, *p* < 0.001, d = 1.75 respectively). For walking conditions, RF H-reflex was higher in post-stroke SKG compared to healthy *Walk* (F_(1,276)_ = 10.8, *p* = 0.004, d = 1.58) and *Walk*↑ (F_(1,351)_ = 4.72,, *p* = 0.044, d = 1.04). RF H-reflex was also higher in healthy *Walk*↑ compared to healthy *Walk* (F_(1,360)_ = 235, t = 15.35, *p* < 0.001, d = 1.61).

No significant correlations were observed between RF EMG activities before stimulation and corresponding H-reflex responses in standing trials for participants with post-stroke SKG (*R* = 0.38, *p* = 0.27) and healthy individuals (*R* = − 0.29, *p* = 0.42).

**Fig. 6:**
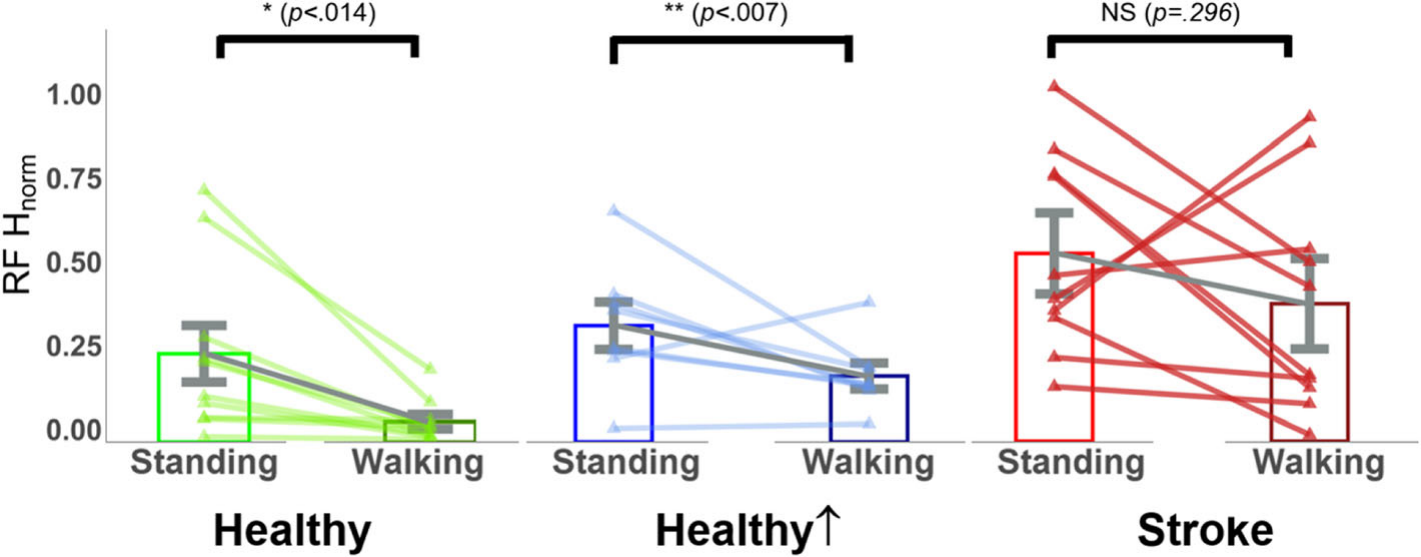
RF H-reflex modulation during walking is absent in post-stroke SKG. RF H-reflex magnitudes were decreased in healthy controls between *Stand* and *Walk*, and *Stand*↑ and *Walk*↑ conditions. There was no significant difference in participants with post-stroke SKG. Triangles represent individual subject data, whereas bars represent mean and 95% confidence intervals of the group

The RF H-reflexes were significantly increased in *Stand* compared to *Walk* (t = 3.23, *p* = 0.014, d = 3.24), and *Stand*↑ compared to *Walk*↑ (t = 3.78, *p* = 0.007, d = 2.86) in healthy controls, whereas no change was observed between *Stand* and *Walk* in participants with post-stroke SKG (t = 1.10, *p* = 0.296, d = 0.73). Figure 6 illustrates these relationships on group and individual levels.

## Discussion

Our previous work used robotic pre-swing knee flexion perturbations in people with post-stroke SKG to address the supposed proximate cause, reduced pre-swing knee flexion velocity [31]. While these perturbations resulted in greater overall knee flexion angle during swing, we observed extension reflective of quadriceps spasticity [40]. Further musculoskeletal modeling analysis agreed with this observation [1], suggesting that knee flexion perturbations elicited RF hyperreflexia. The aim of this study was to determine the influence of RF hyperreflexia on reduced knee flexion in individuals with post-stroke SKG. Instead of using robotic perturbations, we elicited well-controlled H-reflex responses during walking and standing. We distinguished the effects of increased voluntary quadriceps contraction on RF H-reflex modulation by increasing EMG activity in healthy controls during standing and walking. Our results revealed a strong correlation between elevated RF H-reflex at toe-off and reduced peak knee flexion during swing phase in SKG, in agreement with our previous work. We observed no such relation in healthy individuals walking normally or with increased RF activation. The RF H-reflex was modulated in healthy controls between walking and standing, even with increased homonymous muscle activation, whereas no such modulation was observed in post-stroke SKG. The RF H-reflex amplitude was higher during toe-off in post-stroke SKG than healthy individuals with or without increased RF activity. Taken together, these findings suggest new evidence that quadriceps hyperreflexia contributes to gait impairment in post-stroke SKG.

Quadriceps muscle overactivity during pre-swing has been observed in people with post-stroke SKG [18]. The consequences of such activation have been attributed to reduced knee flexion velocity [31] as well as an excessive knee extension moment during pre-swing phase [13] in those with SKG. However, it is unclear whether such excessive quadriceps activity is related to reflexive mechanisms. We observed a strong negative correlation between quadriceps reflex excitability and reduced knee flexion during swing in those with post-stroke SKG. This correlation was independent of reflex excitability during simulated voluntary “overactivity” of the quadriceps in healthy individuals. Further, roughly half of the difference in knee flexion angle in SKG compared to healthy individuals can be explained by increased reflex excitability. These observations suggest that quadriceps reflex excitability may be an important mechanism of SKG. This finding concurs with a previous study in children with cerebral palsy and SKG, who found a correlation between quadriceps spasticity while seated and knee flexion during walking [7]. However, insufficient ankle push-off may be a primary cause of reduced knee flexion velocity based on the observed correlation between reduced push-off and reduced knee flexion velocity in those with SKG [4]. While lower ankle plantarflexion torque certainly contributes to reduced knee flexion [29], reduced plantarflexion torque could be an adaptation to quadriceps spasticity. Further research is needed to determine such complex interactions. Thus, our findings suggest that it is unlikely that poorly timed, graded or coordinated quadriceps activation modulates hyperactive quadriceps monosynaptic reflex, but rather increased reflex excitability is likely an independent mechanism of SKG. Interventions targeted at modulating quadriceps hyperreflexia in SKG can help establish the causality of this relationship.

We found context-dependent RF H-reflex modulation in healthy individuals between standing and walking but no change in RF H-reflexes between standing and walking in SKG. Controls, quadriceps and soleus H-reflex are modulated in a phase- and task-dependent manner [5, 22, 25, 26]. Our results showed H-reflex modulation in healthy individuals between standing and the pre-swing phase of walking, as expected. While additional observations revealed that increased background EMG in the RF elevated RF H-reflex during walking, the increase was substantially less than the increased H-reflex in post-stroke SKG. These results suggest that heightened efferent activity of quadriceps was not responsible for the lack of reflex modulation observed in SKG.

The correlation between increased H-reflex amplitude and decreased knee flexion angle introduces a specific neuromechanical factor that requires further investigation in SKG. Additional mechanisms include heterogeneous abnormal mechanisms such as altered coordination between unaffected and affected knee extensors [17], altered heteronymous reflex pathways influencing knee extensors and ankle flexors [10], and simultaneous involuntary knee extensor and hip adductor activity [12], all of which could have potentially have influenced knee movement in SKG. The evaluation of these mechanisms introduces additional practical challenges such as concurrent nerve stimulation or customized perturbations for specific joint movements during walking. More importantly, the influence of homonymous pathways on individual joint movements during gait is necessary to establish prior to more complex coordinated joint movements governed by heterogeneous neural mechanisms. The revealed relation between elevated monosynaptic quadriceps stretch reflex responses and knee flexion is an important step towards establishing the influence of homonymous pathways in SKG.

This study has practical limitations preventing the accurate imitation of over-active quadriceps in SKG on healthy controls. The increased RF activity in standing and walking was limited to voluntary activation generated by healthy subjects did not accurately represent the influence of altered intrinsic muscle properties [15] and the altered quadriceps activity originating from reduced muscle coordination [6] in SKG. In addition, the resulting knee flexion varied between subjects as opposed to the consistent reduced knee flexion in SKG. The heterogeneous RF H-reflex modulation during walking compared to standing and RF H-reflex hyperexcitability suggests that the quadriceps H-reflex is a prominent factor for reduced knee flexion in SKG.

There are additional limitations that affect interpretation. We estimated the H-reflex amplitude (Fig. 2) while trying to overcome a potential overlap with the M-wave. While this estimation could introduce some variability into the data, it is significantly improved compared to methods used previously [24, 25]. Although all post-stroke participants had SKG, the participant sample showed some heterogeneity in functional ability, previous use of medication, knee flexion, time post-stroke, AFO, and other parameters (Table 1). This heterogeneity, combined with small sample size, would make it difficult to conclusively state the effects these factors on H-reflex modulation. Despite these limitations, we did not observe any trends suggesting these factors, such as use of AFO or previous use of Botox, affects H-reflex size. Furthermore, our results are remarkably consistent between post-stroke individuals. SKG and healthy subjects differed in age (SKG was 19.2 years older). While age does affect H-reflex excitability, it has a dampening effect with age [37] as opposed to increased excitability, which was found in our older SKG group. In this manner, the use of a healthy young cohort provides a more appropriate control condition than age matching.

## Conclusion

Our results suggest that quadriceps hyperreflexia is associated with diminished knee flexion during swing phase in people with post-stroke SKG. Such information could provide a more accurate and quantifiable factor to evaluate the influence of spasticity in post-stroke SKG. Further work is needed to delineate the causal neural mechanisms of this interaction. The implications of this work suggest that interventions targeted at quadriceps hyperreflexia may help elucidate its influence on post-stroke SKG.

## Supplementary information

**Additional file 1: Supplementary Table 1.** Participant information for healthy controls (*N*=10).

## Data Availability

Data supporting the conclusions of this manuscript is included within the manuscript. The data collected in this study are available from the corresponding author on reasonable request.

## Abbreviations

SKG: Stiff-Knee gait
RF: Rectus femoris
M_max_: Maximum M-wave
AL: Adductor longus
GMed: Gluteus medius
TFL: Tensor fasciae latae
GMax: Gluteus maximus
MH: Medial hamstrings
VM: Vastus medialis
