# Supplementary material for: Rectus femoris hyperreflexia contributes to Stiff-Knee gait after stroke
Source: J Neuroeng Rehabil. 2020 Aug 26;17:117. doi: 10.1186/s12984-020-00724-z (PMC7448457; doi:10.1186/s12984-020-00724-z)
Supplement: Supplementary file 1 — Additional file 1: Supplementary Table 1. Participant information for healthy controls (N=10). [file 12984_2020_724_MOESM1_ESM.docx]

| **Supplementary Table 1. Data for 10 healthy participants** | | | | | | |
| --- | --- | --- | --- | --- | --- | --- |
| Subject no | Age | Gender | Side | Weight (kg) | Peak knee flexion (degree) | |
|  |  |  |  |  | Baseline | Feedback |
| 1 | 23 | F | L | 89 | 61.4 | 36.9 |
| 2 | 23 | F | R | 59 | 61.3 | 42.9 |
| 3 | 56 | F | R | 57 | 53.7 | 35.1 |
| 4 | 43 | M | L | 64 | 48.7 | 36.7 |
| 5 | 38 | M | L | 73 | 51.5 | 45.5 |
| 6 | 21 | M | R | 60 | 43.7 | 58.9 |
| 7 | 22 | M | R | 87 | 49.2 | 54.3 |
| 8 | 36 | M | L | 93 | 46.1 | 50.0 |
| 9 | 30 | M | R | 92 | 50.7 | 27.9 |
| 10 | 26 | F | R | 55 | 46.7 | - |
| Mean | 32 |  |  | 72.9 | 51.3 | 42.8 |
| SD | 11 |  |  | 15.8 | 6.0 | 9.5 |
| *Side indicates the selected leg segment for the nerve stimulation to induce H-reflex response  * Peak knee flexion column indicates the mean±sd of maximum knee flexion angle measures of the stimulated sides during walking. Baseline and feedback indicates stimulation without and with increased RF muscle contraction with EMG feedback respectively. | | | | | | |
